# Exploration of *Giardia* small nucleolar RNAs (snoRNAs) and their possible microRNA derivatives

**DOI:** 10.1017/S003118202400060X

**Published:** 2024-05

**Authors:** Francisco Alejandro Lagunas-Rangel

**Affiliations:** Department of Surgical Sciences, Uppsala University, BMC Box 593, Husargatan 3, 751 24, Uppsala, Sweden

**Keywords:** Argonaute, C/D-box snoRNAs, H/ACA-box snoRNAs, miRNA biogenesis machinery, parasite

## Abstract

Small nucleolar RNAs (snoRNAs) are short non-coding RNAs that are abundant in the nucleoli of eukaryotic cells and play a crucial role in various aspects of ribosomal RNA (rRNA) maturation, including modifications such as 2′-O-methylation or pseudouridylation. On the other hand, *Giardia duodenalis* is a microaerophilic, flagellated, binucleate protozoan responsible for causing giardiasis. Although numerous snoRNAs have been detected in *Giardia*, their investigation remains limited. Nevertheless, they have been found to play a crucial role in the rRNA precursor processing pathway and influence other cellular functions. In addition, it has been proposed that some microRNAs are generated from these snoRNAs through excision by the *Giardia* endoribonuclease Dicer. These microRNAs are believed to contribute to the regulation of antigenic variation, which allows the parasite to evade the host immune response. Specifically, they play a role in modulating variant-specific surface proteins (VSPs) and other cysteine-rich surface antigens (CSAs). The main objective of this study was to bring together the available data on snoRNAs in *Giardia*, uncovering their functions in various processes and their importance on a global scale. In addition, the research delved into potential microRNAs speculated to originate from snoRNAs, exploring their impact on cellular processes.

## Graphical abstract

**Figure:**
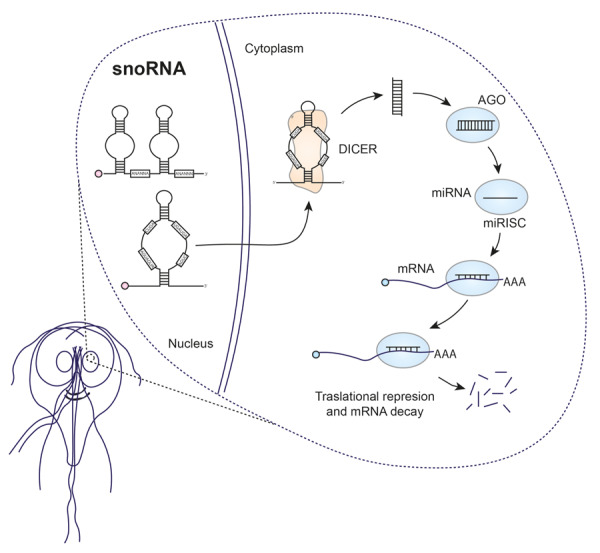

## Introduction

Small nucleolar RNAs (snoRNAs) are short non-coding RNAs with a typical length ranging from 60 to 300 nucleotides. They are abundant in the nucleoli of eukaryotic cells and play a key role in several aspects of ribosomal RNA (rRNA) maturation (Huang *et al*., 2022). Most snoRNAs direct modifications, such as 2′-O-methylation or pseudouridylation, at specific positions within the rRNA, while others are involved in critical cleavage events that are integral steps in the complex rRNA precursor processing pathway (Kolev and Ullu, 2009).

Although all classes of snoRNAs interact with various proteins to form small nucleolar ribonucleoproteins (snoRNPs) with catalytic functions, it is the base-pairing interactions of the snoRNA with the target rRNA that guide the modifying enzymes (nucleolar protein 1 [NOP1] or centromere-binding factor 5 [CBF5]) to the precise sites of modification. This intricate mechanism ensures the precise positioning of modifications within the rRNA molecule, underscoring the critical role of snoRNAs in the organization of rRNA maturation (Kolev and Ullu, 2009; Yan *et al*., 2019).

On the other hand, *Giardia duodenalis* is a microanaerobic flagellated binucleate protozoan with an extraordinarily compact genome of 12.6 Mb (Xu *et al*., 2020). This microorganism is characterized by a reduction in the number of components used in several cellular processes. In particular, it lacks some organelles typical of eukaryotic cells, such as the classical Golgi apparatus, peroxisomes and respiratory mitochondria (Cernikova *et al*., 2018). Indeed, *Giardia* was for a long time thought to lack a nucleolus and was considered as a ‘primitive’ or ‘early branching lineage’ organism (Lagunas-Rangel, 2023, 2024). It is now more widely accepted that the unique characteristics of *Giardia* are the result of reductive evolutionary processes associated with its transition to obligate parasitism (Lloyd and Harris, 2002; Burki *et al*., 2020).

Notably, *Giardia* is the causative agent of giardiasis, a disease responsible for more than 300 million cases of diarrhoeal disease worldwide, especially prevalent in developing and low-income countries (Cernikova *et al*., 2018). Although *Giardia* infections can sometimes be asymptomatic, patients may also present with symptoms such as nausea, epigastric pain and, in certain cases, weight loss. Furthermore, in children, the infection increases the risk of malabsorption syndrome, a major medical problem (Einarsson *et al*., 2016; Allain and Buret, 2020).

The life cycle of *Giardia* is relatively simple and consists of 2 main phases: the trophozoite, which is the vegetative and motile form, and the cyst, which is environmentally resistant and highly infective (Lagunas-Rangel *et al*., 2021). Upon ingestion by the host, the cysts are activated in the gastrointestinal tract by the acidity of the stomach and subsequent exposure to bile and trypsin in the duodenum. This activation leads to the release of motile trophozoites in the upper small intestine. In this region, characterized by abundant nutrients and low oxygen levels, the trophozoites thrive, adhering to the intestinal villi with their adhesive disc and resisting peristalsis. As trophozoite density increases and they migrate to the lower intestine, they encounter various environmental changes, such as decreased cholesterol levels, increased pH, and higher concentrations of bile and lactic acid. These conditions cause a subset of trophozoites to differentiate and transform into infectious cysts. These cysts are excreted in the feces, serving as a reservoir for new infections (Barash *et al*., 2017; Lagunas-Rangel *et al*., 2021*b*).

Several snoRNAs have been identified in *Giardia*, some of which share similarities with those found in human cells and yeast, while others are unique to the parasite (Yang *et al*., 2005). These snoRNAs have been little studied, but have been implicated in the rRNA precursor processing pathway and other cellular functions (Niu *et al*., 1994; Yang *et al*., 2005; Saraiya and Wang, 2008; Kolev and Ullu, 2009; Saraiya *et al*., 2011; Huang *et al*., 2012; Li *et al*., 2012). In addition, it has been suggested that some small RNAs are generated from these snoRNAs through excision by the *Giardia* endoribonuclease Dicer. These small RNAs apparently play a role in the regulation of variant-specific surface proteins (VSPs) and other cysteine-rich surface antigens (CSAs) (Saraiya and Wang, 2008; Saraiya *et al*., 2011, 2014; Huang *et al*., 2012; Li *et al*., 2012). This regulation could be crucial for the ability of *Giardia* to undergo antigenic variation and evade the immune response of the infected host (Gargantini *et al*., 2016).

In this way, the aim of this study was to compile existing information on snoRNAs in *Giardia* and to describe their involvement in cellular processes and their relevance. Furthermore, potential miRNAs speculated to originate from snoRNAs and their roles in cellular processes and the molecular mechanisms involved were examined.

## snoRNAs

**Figure 1.**
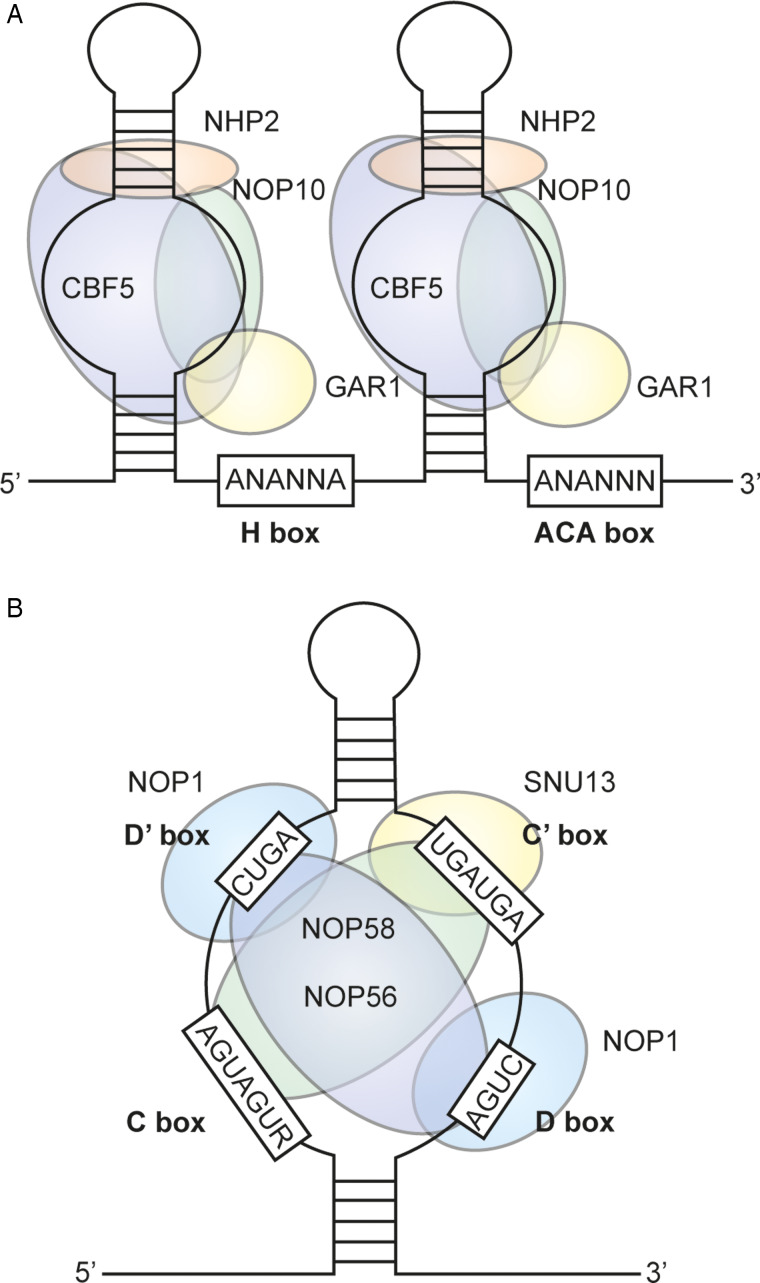
Schematic representation of the main snoRNAs. (A) H/ACA-box snoRNAs. (B) C/D-box snoRNAs.

In humans, snoRNAs reside mainly in the intronic regions of a multitude of genes, encompassing both coding and non-coding sequences. Their classification depends on the conserved sequence motifs that dictate their interactions with different sets of proteins. Specifically, snoRNAs are usually classified into three main groups: H/ACA-box snoRNAs (Fig. 1A), C/D-box snoRNAs (Fig. 1B) and small Cajal RNAs (scaRNAs) (Huang *et al*., 2022).

H/ACA-box snoRNAs typically span between 60 and 75 nucleotides and feature a pseudouridylation pocket region responsible for isomerizing uridine residues on the substrate RNA (Bortolin, 1999). Within this process, yeast H/ACA-box snoRNAs interact with phosphorylated proteins such as CBF5, NOP10, glycine/arginine-rich protein 1 (GAR1) and high mobility group-like nuclear protein 2 (NHP2), with CBF5 playing a key role as a catalytic protein in pseudouridylation (Meier, 2006). Eukaryotic H/ACA-box snoRNAs have 2 conserved sequences: the H-box, characterized by an ANANNA consensus sequence, located downstream of the first hairpin, and the ACA-box, consisting of an ACA trinucleotide, located downstream of the second hairpin (Ganot *et al*., 1997).

Meanwhile, eukaryotic C/D-box snoRNAs are typically between 70 and 120 nucleotides in length. These snoRNAs feature 2 conserved sequences known as C-box and D-box. The C-box, located at the 5′ end of the snoRNA molecule, is formed by the RUGAUGA nucleotides. In contrast, the D-box, located at the 3′ end, is formed by CUGA nucleotides (Kiss-László *et al*., 1996). These elements rely on base-pairing interactions to fold into a specific structure known as ‘kink-turn’. This structural motif is recognized in yeast by 13 KDa small nuclear ribonucleoprotein-associated protein (SNU13), which subsequently recruits NOP1 (also known as fibrillarin or FBL), NOP58 and NOP56 to facilitate the 2′-O-methylation modification process, where NOP1 is the main catalyst of the reaction (Baldini *et al*., 2021).

Finally, scaRNAs represent a subtype of snoRNAs located in Cajal bodies (CBs). CBs are membraneless organelles composed predominantly of proteins and RNA, located within the nucleus of proliferative cells and metabolically active neurons. Like other snoRNAs, they adhere to the C/D-H/ACA classification system, although some scaRNAs show a combination of C/D and H/ACA structures (Bratkovič and Rogelj, 2014).

## Giardia snoRNAs

In *Giardia*, 20 snoRNAs were initially identified, including 16 C/D-box snoRNAs and 4 H/ACA-box snoRNAs (Table 1) (Yang *et al*., 2005). Subsequently, additional snoRNAs were identified by bioinformatics and experimental methods (Saraiya and Wang, 2008; Chen *et al*., 2011; Huang *et al*., 2012; Li *et al*., 2012). Unlike vertebrates, where most snoRNAs are encoded within introns and processed by exonucleases (Kiss and Filipowicz, 1995; Maxwell and Fournier, 1995), *Giardia* snoRNAs are organized differently. They are organized as independently transcribed clusters, with known snoRNAs encoded in the short intergenic regions between protein-coding genes (Yang *et al*., 2005; Chen *et al*., 2007). Each region contains a single snoRNA (Luo *et al*., 2009). This may be because *Giardia* has only a few intron-containing genes (Seabolt *et al*., 2023). Currently, it remains unclear whether these snoRNAs are independently transcribed, although there is evidence to suggest this possibility (Niu *et al*., 1994; Yang *et al*., 2005). Although experimental identification of the promoters of all these snoRNAs is currently unavailable, a common feature observed is the presence of 1 or 2 A-T-rich elements, typically spanning 10–20 base pairs in length, located approximately 70 base pairs upstream of the snoRNA genes. This shared feature aligns with the promoter structure found in protein-coding genes in *Giardia* (Yang *et al*., 2005; Lagunas-Rangel *et al*., 2021*a*).

**Table 1.** First reported *Giardia* snoRNAs

Name	Class	snoRNA length (nt)	Relative abundance	rRNA target	Homologues			miRNA
					Yeast	Plant	Vertebrate	
GLsR1	C/D box	77	+ + +	16S-Cm1325	snR70	AtU43	U43	miR6
GLsR2	C/D box	104	+ +	16S-Cm334	U14	AtU14	U14	miR5
GLsR3	C/D box	79	+ +	16S-Gm1178				
GLsR4	C/D box	61	+	23S-Gm2040	snR67	AtsnoR35	U31	
GLsR5	C/D box	89	+ +	23S-Gm1880	snR190		MBII-99	
GLsR6	C/D box	51	+ +	23S-Gm2234	snR38	AtsnR38	snR38	
GLsR7	C/D box	60	+	23S-Am1741	snR63			
GLsR8	C/D box	67	+	23S-Am525	snR39/59	AtU51	U51/U32	miR10
GLsR9	C/D box	79	+ + +	23S-Am414				
GLsR10	C/D box	65	+	23S-Am2456				
GLsR11	C/D box	142	+	23S Cm893				
GLsR13	C/D box	100	+					
GLsR14	C/D box	73	+					
GLsR15	C/D box	61	+ +					
GLsR16	C/D box	77	+ +					miR3
GLsR17	C/D box	144	+ + +					miR2
GLsR18	H/ACA box	101	+ +	23S-2345		AtsnoR74		
GLsR19	H/ACA box	176	+	23S-1801				
GLsR20	H/ACA box	108	+	23S-2248 23S-2365	snR34 snR37	AtU65	U65	
GLsR21	H/ACA box	107	+					

The 2,2,7-trimethylguanosine cap has been identified in both small nucleolar and nuclear RNAs within the *Giardia* framework, exerting significant influence on crucial RNA processes, including stability, splicing and translation efficiency (Lagunas-Rangel *et al*., 2021). The *Giardia* paralogs of human trimethylguanosine synthase (TGS1), termed TGS1 and TGS2, function as enzymes responsible for the conversion of 7-methylguanosine RNA caps to the 2,2,7-trimethylguanosine cap structures of snoRNAs. This conversion is achieved by catalysis of methyl transfer from S-adenosylmethionine (SAM) in a sequential process of both paralogs (Hausmann and Shuman, 2005).

Some *Giardia* snoRNAs share homology and cellular localization with homologues found in yeast, plants or vertebrates, while others are unique to *Giardia*. Interestingly, the latter are located in conserved regions of the rRNA where no 2′-O-methylation sites have been described in other eukaryotes (Yang *et al*., 2005; Luo *et al*., 2009). Hence, it could be suggested that *Giardia* rRNA undergoes unique modifications or, alternatively, that these snoRNAs could perform other functions. *Giardia* snoRNAs show a higher degree of similarity with their homologues identified in other protozoa such as *Dictyostelium discoideum* and *Plasmodium falciparum*, fungi and some metazoa, but contrast sharply with those found in protozoa of the phylum Euglenozoa (Luo *et al*., 2009). Most *Giardia* C/D-box snoRNAs maintain the typical structure found in other organisms, serving as single guide snoRNAs. They are characterized by 2 conserved motifs known as C and D boxes, along with 2 variable structural motifs termed D’ and C’ boxes, and a functional element generally spanning 10–21 nucleotides located upstream of the D or D’ boxes. Similarly, *Giardia* H/ACA-boxed snoRNAs exhibit a characteristic structure with 2 double-stemmed hairpins, a conserved H-box and an ACA-box (Yang *et al*., 2005; Luo *et al*., 2009).

Regarding proteins associated with *Giardia* snoRNAs, orthologs of CBF5 (GL50803_16311), NOP10 (GL50803_8242), GAR1 (GL50803_8794) and NHP2 (GL50803_13926) have been identified for H/ACA-box snoRNAs. Likewise, orthologs of SNU13 (GL50803_11287), NOP1 (GL50803_97219), NOP58 (GL50803_5359) and NOP56 (GL50803_10577) have been identified in the *Giardia* genome for C/D-box snoRNAs. Furthermore, *Giardia* snoRNAs have been observed to interact with many other proteins (Ghosh *et al*., 2001).

Post-transcriptional modifications of *Giardia* rRNA nucleotides have been observed, which exhibit unique characteristics that do not always match those found in humans or other organisms. In particular, 2’-O-methylation or pseudouridylation modifications have been identified in all *Giardia* rRNA subunits, 18S, 28S and 5,8S (Hiregange *et al*., 2022). These modifications suggest the involvement of snoRNAs in the regulation of rRNA maturation and functionality. In this context, *Giardia* H/ACA-box snoRNAs are predicted to guide pseudouridylation at specific sites within the 18S and 28S subunits. Meanwhile, *Giardia* C/D-box snoRNAs have been observed to be associated with 2’-O-ribose methylation sites in *Giardia* rRNAs, mainly targeting the 28S sequence (Yang *et al*., 2005)

## *Giardia* miRNA biogenesis machinery

**Figure 2.**
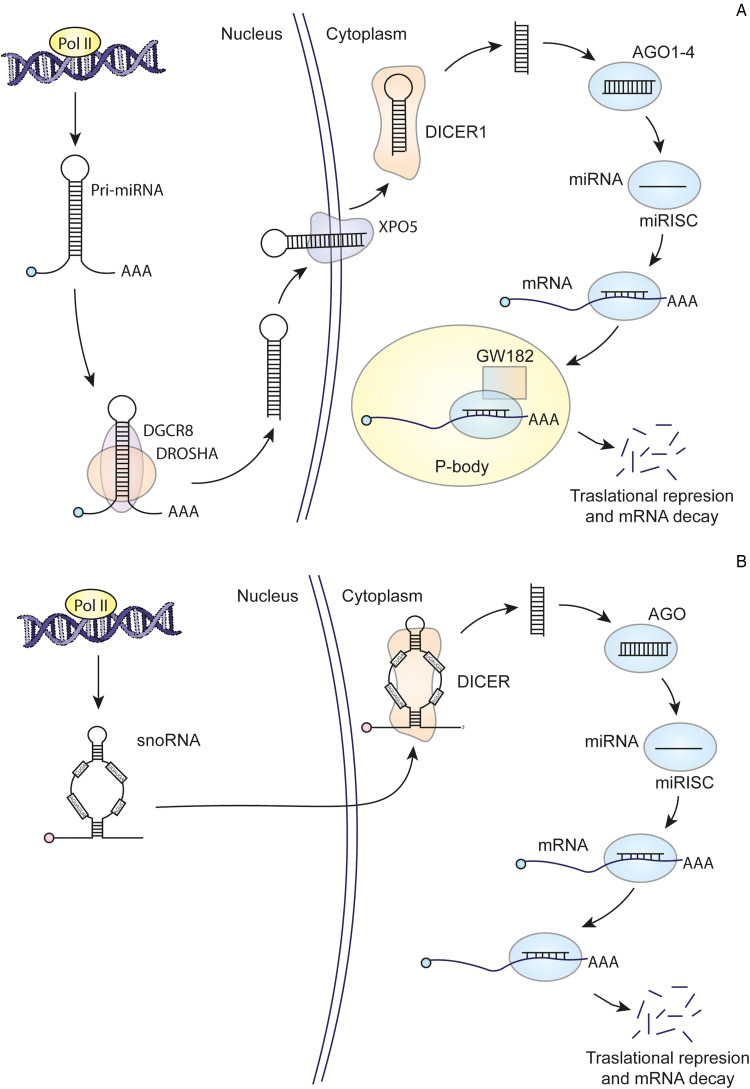
Comparative miRNA biogenesis between humans (A) and *Giardia* (B).

Since 1994, it has been suggested that some small RNAs could originate from *Giardia* snoRNAs (Niu *et al*., 1994). However, for a considerable period it was believed that microRNAs (miRNAs) did not exist in *Giardia*, causing the idea to be overlooked until recent research revived interest in this area. In humans, the canonical pathway of miRNA biogenesis involves several key steps. First, double-stranded RNA-specific endoribonuclease (DROSHA), a protein belonging to the RNase III family, processes long primary transcripts into hairpin miRNA precursors. DROSHA acts in a complex with the double-stranded RNA (dsRNA)-binding protein DGCR8. The resulting pre-miRNA is transported to the cytoplasm by exportin-5 (EXO5). Subsequently, DICER, an RNase III-type enzyme, plays a crucial role. DICER cleaves dsRNAs or RNA hairpins, generating small RNA duplexes. These duplexes are then loaded onto an argonaute (AGO) family protein, forming the RNA-induced silencing complex (RISC) (Fig. 2) (Ha and Kim, 2014). The AGO protein, together with the miRNA guide strand, binds to the target transcripts, causing their silencing or degradation. In addition, in certain organisms such as fungi, plants and nematodes, an additional component known as RNA-dependent RNA polymerase (RdRp) is required for RNA interference (RNAi) production. RdRp is involved in the amplification of the RNAi response through the generation of secondary siRNA or miRNA (Kolev and Ullu, 2009).

*Giardia* possesses certain components of the miRNA biogenesis machinery, including DICER (GL50803_103887), AGO (GL50803_002902) and RdRp (GL50803_102515), but lacks or has not been identified DROSHA or EXO5 (Li *et al*., 2011). *Giardia* DICER shares essential features with DICER proteins from other organisms, such as the Piwi Argonaute Zwille tandem domains (PAZ) and RNase III. However, it lacks certain elements typically present in other DICER proteins, such as the N-terminal DExD/H helicase, the C-terminal double-stranded RNA-binding domain (dsRBD) and extended interdomain regions. This magnesium-dependent enzyme processes dsRNA from the helical end, producing fragments of a typical length of 25–27 nucleotides. Despite its efficient processing capacity, it shows low affinity for its small RNA product and exhibits multiple turnover kinetics (MacRae *et al*., 2006). Significantly, *Giardia* DICER has been observed to efficiently process snoRNAs into miRNAs *in vitro* (Li *et al*., 2012). It also generates products from rRNA or transfer RNA (tRNA) (Li *et al*., 2011, 2012). Meanwhile, *Giardia* AGO is involved in binding to the 7-methylguanosine (m7G) cap of mRNAs, thus competing with *Giardia* eIF4E (GL50803_17261). Notably, down-regulation of AGO in *Giardia* trophozoites resulted in growth inhibition, highlighting the importance of this protein in various biological processes of the parasite. Structurally, *Giardia* AGO conforms to the typical domain arrangement observed in AGO family proteins, with PAZ and PIWI domains. It is assumed to possess endonuclease activity, given the presence of essential residues in the active site (DDH) within the PIWI domain. This suggests that the presence of phosphate and hydroxyl groups in an RNA molecule, rather than sequence specificity, is crucial for its function (Li *et al*., 2011).

## miRNAs derived from snoRNAs

Several studies, encompassing both bioinformatic predictions and experimental investigations, indicate that miRNAs derived from *Giardia* snoRNAs have important biological implications in this parasite (Saraiya and Wang, 2008; Saraiya *et al*., 2011; Huang *et al*., 2012; Li *et al*., 2012). A miRNA named miR2, whose origin has been predicted from the *Giardia* snoRNA GlsR17, has been implicated in the regulation of VSPs and other CSAs. This regulation is achieved through the interaction of miR2 with the 3’-unstranlated region (UTR) of the respective mRNAs (Saraiya and Wang, 2008).

Antigenic variation is a crucial process for *Giardia* in the perpetuation of infection. This phenomenon involves the periodic replacement of a specific variant of VSP family, which is expressed on the surface of proliferating trophozoites, with another variant. This cycle allows *Giardia* to efficiently evade the host immune response. The parasite genome contains approximately 200 VSP genes, which provide the genetic repertoire sufficient for this process to be efficient (Gargantini *et al*., 2016).

Other miRNAs with similar functions are miR6 and miR10, derived from the snoRNAs GlsR1 and GlsR8, respectively. Both miRNAs present a hairpin structure, with the miRNA sequence located at the 3′ end of 1 of the 2 stem arms. A binding site for miR6 was identified in the 3′-UTRs of 44 VSP genes, whereas a binding site for miR10 was found in the 3′ end of 159 VSP open reading frames. Notably, 33 VSP genes contain binding sites for both miR6 and miR10. Among them is VSP1267 (also referred to as VSP-98.1 or GL50803_112208), which not only harbours target sites for both miRNAs, but has also been experimentally shown to have cooperative actions between the 2 miRNAs (Li *et al*., 2012). Similarly, a cooperative action between miR2 and miR10 can be observed, leading to inhibition of VSP213 (GL50803_114122) (Li *et al*., 2012). This suggests that multiple snoRNA-derived miRNAs may play an active and substantial role in regulating *Giardia* antigenic variation, potentially acting in concert.

It has also been observed that certain miRNAs can show species specificity. For example, a 24-nucleotide miRNA, known as miR4, processed by DICER and AGO, and derived from an unannotated ORF (GL50803_92663) is specific to *Giardia* WB and regulates VSP proteins, including VSP-213 (GL50803_114122) (Saraiya *et al*., 2011). Thus, miRNAs would complement currently known epigenetic regulatory mechanisms to regulate antigenic variation (Lagunas-Rangel and Bermúdez-Cruz, 2019). In addition to regulating VSP expression, snoRNA-derived miRNAs have been found to target mRNAs of other proteins, such as NEK kinases (Li *et al*., 2011). *Giardia* is known for its significant expansion of the NEK protein family, which comprises approximately 198 proteins, constituting about 4% of its proteome. This extensive presence of NEK kinases in *Giardia* implies a sophisticated mechanism of regulation, especially crucial during the cell cycle (Manning *et al*., 2011; Lagunas-Rangel *et al*., 2021*b*). A study investigating miRNAs in some flagellated protozoan parasites, including *Giardia*, revealed that only 5 miRNA families are shared among them, such as LET-7, MIR-1, MIR-122, MIR-3596 and MIR167_1 (Huang *et al*., 2012). LET-7 has been shown to regulate macrophage immune functions in *Echinococcus multilocularis* and *Taenia pisiformis* (Jin *et al*., 2021; Wang *et al*., 2021).

## Conclusions and perspectives

**Figure 3.**
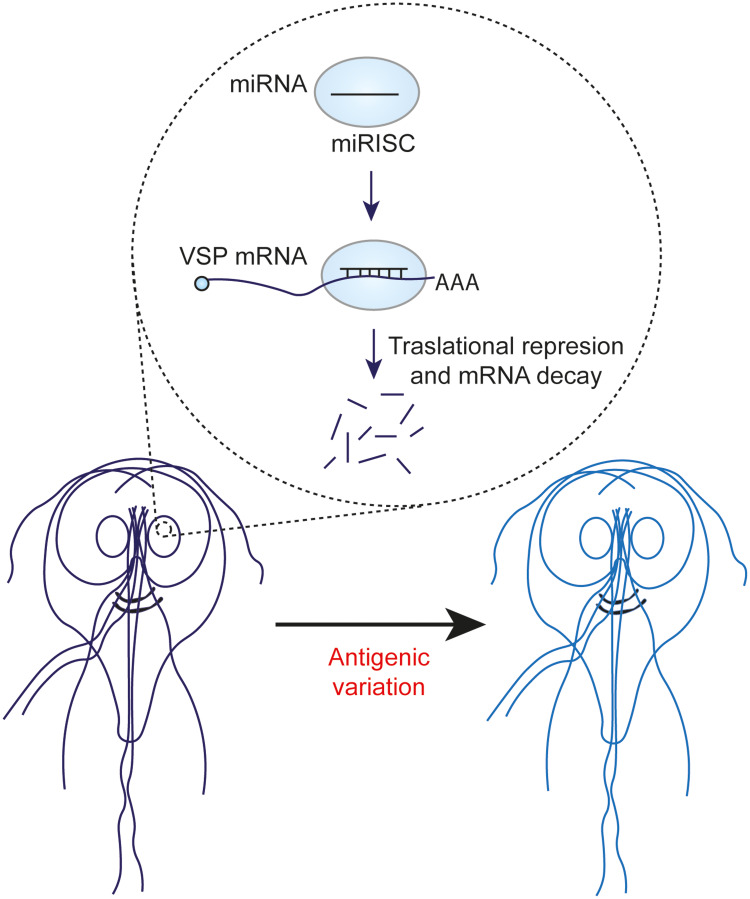
miRNAs derived from snoRNAs could influence the antigenic variation of *Giardia*.

*Giardia* snoRNAs, although relatively understudied, have been shown to play crucial roles in several cellular processes of the parasite. Their main function is to facilitate rRNA maturation by orchestrating modifications such as 2′-O-methylation or pseudouridylation. In addition, there is increasing evidence that *Giardia* snoRNAs may have broader activities than rRNA processing. Moreover, there is increasing speculation that processing of these snoRNAs by *Giardia* DICER gives rise to miRNAs, which could influence critical aspects of parasite biology, such as antigenic variation (Fig. 3). These miRNAs appear to regulate the expression of multiple VSP transcripts. In addition, they are predicted to target NEK protein kinases, which are crucial for various cellular processes, including cell division. However, it is essential to confirm the presence of miRNAs in *Giardia* and elucidate their specific functions through further experimental studies. Harnessing knowledge of snoRNAs and potential miRNAs in *Giardia* could pave the way for the development of new therapeutic strategies for giardiasis.

In the future, there is potential for in-depth investigation of *Giardia* snoRNAs along several dimensions, including their sequence characteristics, promoter regions and the regulatory mechanisms governing their transcription. In addition, we must deepen our understanding of their role in post-translational modification and/or facilitation of rRNA processing. By delving deeper into these functions, we may uncover pathways amenable to manipulation for targeted therapeutic interventions. Of particular importance is understanding the intricate processing of snoRNAs, especially given the absence of key proteins such as DROSHA and EXO5 in *Giardia*. This knowledge gap provides an opportunity to elucidate the precise mechanisms by which snoRNAs give rise to their derived miRNAs. Furthermore, it is crucial to identify the full spectrum of targets of these miRNAs and to understand their role in the regulation of VSP mRNA degradation. This knowledge could pave the way for the development of strategies aimed at preventing antigenic variation of the parasite, thus increasing the efficacy of the immune system to eliminate it. In particular, the influence of AGO on *Giardia* cell growth suggests its potential as a therapeutic target, opening avenues to explore AGO-directed interventions. This is particularly crucial given the increasing prevalence of *Giardia* strains resistant to current antiparasitic drugs. Investigating the functions of these miRNAs may offer new avenues to effectively combat giardiasis.

## Data Availability

No datasets were generated or analysed during the current study.
